# Safety and Efficacy of Bis-Glyceryl Ascorbate (Amitose DGA) to Prevent Hand-Foot Skin Reaction in Patients With Renal Cell Carcinoma Receiving Sunitinib Therapy: Protocol for a Phase I/II, Uncontrolled, Single-Arm, Open-Label Trial

**DOI:** 10.2196/14636

**Published:** 2019-08-12

**Authors:** Kazuhiro Yamamoto, Takeshi Ioroi, Kenichi Harada, Satoshi Nishiyama, Chikako Nishigori, Ikuko Yano

**Affiliations:** 1 Department of Pharmacy Kobe University Hospital Kobe Japan; 2 Department of Urology Graduate School of Medicine Kobe University Kobe Japan; 3 Division of Dermatology, Department of Internal Related Graduate School of Medicine Kobe University Kobe Japan

**Keywords:** hand-foot skin reactions, renal cell carcinoma, tyrosine kinase inhibitors, ascorbic acid derivative

## Abstract

**Background:**

Hand-foot skin reaction (HFSR) is a serious side effect induced by multiple-tyrosine kinase inhibitors (TKIs). HFSR can cause treatment interruption or decreased dosing. HFSR also markedly decreases quality of life and is associated with the therapeutic efficacy of multiple-TKIs. Therefore, the management and prevention of HFSR is an important issue; however, an effective method for its prevention has not been established. Specific ascorbic acid derivatives can reverse multiple-TKI-induced keratinocyte growth and pathological changes in vitro.

**Objective:**

This study was designed to evaluate the safety of bis-glyceryl ascorbate (Amitose DGA), a novel, hydrosoluble, and moisturizing ascorbic acid derivative, in patients with renal cell carcinoma (RCC) receiving sunitinib therapy. This study was also designed to evaluate Amitose DGA’s preventive efficacy for sunitinib-induced HFSR.

**Methods:**

This is a Phase I/II, single-center, uncontrolled, single-arm, open-label trial. We will recruit a total of 30 patients with RCC receiving sunitinib therapy, with a 2-week-on and 1-week-off schedule. The participants will apply Amitose DGA-containing cream over both palmar and plantar surfaces within two treatment cycles (ie, 6 weeks) of sunitinib in combination with a general moisturizing agent, in addition to standard-of-care processes. Safety assessments will include dermatological abnormalities, clinical laboratory tests, and incidence of adverse events. Efficacy assessments will include development of HFSR and therapeutic outcomes associated with sunitinib.

**Results:**

Recruitment to the study began in August 2017 and is ongoing in Japan. To date, 21 subjects have been recruited. Study completion is expected in 2021.

**Conclusions:**

This is the first clinical study of Amitose DGA-containing cream in patients with RCC who are receiving sunitinib therapy. The single-center, single-arm, open-label design was selected to maximize subject exposure and increase the likelihood of achieving our study endpoints. The results will provide valuable and preliminary evidence of the effects of Amitose DGA-containing cream on HFSR.

**Trial Registration:**

UMIN Clinical Trials Registry UMIN000027209; https://upload.umin.ac.jp/cgi-open-bin/ctr /ctr_view.cgi?recptno=R000031174

**International Registered Report Identifier (IRRID):**

DERR1-10.2196/14636

## Introduction

### Background

In recent years, several types of novel multiple-tyrosine kinase inhibitors (TKIs) against renal cell carcinoma (RCC) have been developed and applied in clinical practice settings [1]. Sunitinib, a multiple-TKI, is an especially important first-line therapy option for patients with metastatic RCC and is the most commonly used agent in clinical settings [2,3]. However, multiple-TKIs can cause serious side effects, which might cause treatment interruption or decrease of dose [4,5]. Hand-foot skin reaction (HFSR) is one such side effect. HFSR is a common and characteristic adverse reaction to multiple-TKIs [6,7] and develops as hyperkeratosis and redness on the palmar and plantar surfaces [7-9]. These side effects can cause walking difficulties and depressed holding and gripping function by the hands, potentially decreasing quality of life; in addition, there is a well-known association between development of HFSR and the therapeutic efficacy of multiple-TKIs [10,11]. Therefore, the management and effective prevention of HFSR has the potential to improve quality of life and the therapeutic outcomes of multiple-TKI treatments [10,12]. Although multiple-TKI-induced HFSR is recognized as a serious problem in clinical practice, effective methods for prevention have not been established.

We recently reported on ascorbyl-2-phosphate magnesium (P-VC-Mg), a highly permeable ascorbic acid derivative; we found that it could relieve multiple-TKI-induced keratinocyte growth inhibition and pathological changes in human keratinocyte cells in a 3D skin model, mediating signal transducer and activator of transcription 3 phosphorylation levels [13,14]. This report suggested that specific ascorbic acid derivatives can prevent multiple-TKI-induced HFSR. Because ascorbic acid derivatives are widely used within cosmetic preparations, it may be possible to establish additional safety parameters.

Bis-glyceryl ascorbate (Amitose DGA) is a novel hydrosoluble and moisturizing ascorbic acid derivative produced by binding ascorbic acid to glycerin. It is easily formulated as an emulsion and gel cosmetic because it is nonionic and has higher permeability and stability compared to other ascorbic acid derivatives. Our preliminary investigation used various ascorbic acid derivatives for keratinocyte growth inhibition with sunitinib. Amitose DGA showed the highest preventive effects among all the products examined, including P-VC-Mg. This effect was attributable to higher cellular translocation and environmental stability of Amitose DGA. There are various reports about the anti-inflammatory effects of ascorbic acid derivatives in healthy or diseased skin and keratinocytes [15-17]; however, its effects on HFSR induced by anticancer agents have not been reported. In spite of this, we consider Amitose DGA to be a strong candidate for HFSR prophylaxis.

### Study Objectives

The primary objectives of this study are to evaluate the dermatological safety of Amitose DGA in patients with RCC who are receiving sunitinib therapy (Phase I) and to determine its prophylaxis efficacy in sunitinib-induced HFSR (Phase II). The secondary objectives are as follows:

1. Evaluate hematological abnormality of Amitose DGA-containing cream
2. Evaluate the effects of Amitose DGA-containing cream on severe sunitinib-induced HFSR
3. Evaluate the effects of Amitose DGA-containing cream on therapeutic outcomes related to sunitinib

## Methods

### Study Design

**Figure 1:**
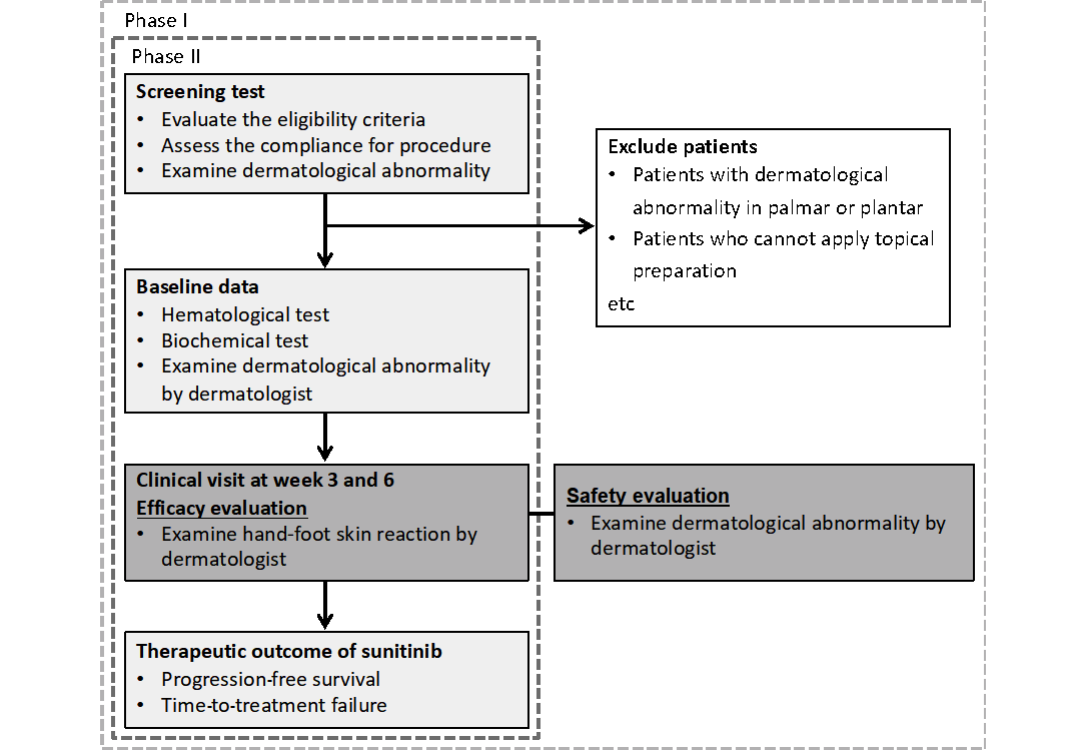
Flowchart of the study design.

This study was designed to verify whether Amitose DGA safely and effectively prevents multiple-TKI-induced HFSR. This is an ongoing Phase I/II, single-center, uncontrolled, single-arm, open-label clinical trial to evaluate the safety and efficacy of Amitose DGA-containing cream as a prophylaxis for sunitinib-induced HFSR. Figure 1 summarizes the study design.

### Study Location and Population

Subjects are being recruited from Kobe University Hospital, Kobe, Japan. The population of this study includes patients with RCC who are receiving sunitinib therapy at the hospital.

### Ethics Approval and Consent to Participate

This study was approved by the Ethics Committee at Kobe University Hospital (approval number: 290015) on August 22, 2017. Each participant will sign an informed consent form, which is worded in lay terms, following a comprehensive explanation of study procedures by a research collaborator; study-related risks and benefits will also be explained before participating in the study. To maximize the opportunity for free and informed consent, while respecting privacy and confidentiality, the informed consent process will take place privately. Potential participants will not be invited to join the study if the clinical research coordinator is not able to adequately explain the study and obtain informed consent. The study will be conducted in compliance with the protocol; regulatory requirements; Ethical Guidelines for Medical and Health Research Involving Human Subjects published by the Japanese Ministry of Health, Labour and Welfare; and the ethical principles of the latest version of the Declaration of Helsinki. Each substantial protocol amendment will receive approval by the Ethics Committee prior to implementation.

### Inclusion and Exclusion Criteria

We will include individuals capable of providing informed consent, aged 20 years or older, with histologically diagnosed RCC, receiving sunitinib therapy, with or without prior molecular targeted therapy, and before or after nephrectomy. All patients have an Eastern Cooperative Oncology Group Performance Status of 0-2 and are expected to survive for more than 12 weeks at screening. Finally, all the included patients will be determined to exhibit higher compliance for applying the investigational cream, attending clinical visits, undergoing laboratory tests, and keeping a personal dairy based on the study protocol. We will exclude patients with dermatological abnormalities of the palmar or plantar surfaces; those who use topical medications on the palmar or plantar surfaces, except for heparinoid or urea-containing cream; those who are unable to apply the heparinoid or urea-containing cream to the palmar or plantar surfaces; those with grade 1 or higher HFSR based on the Common Terminology Criteria for Adverse Events (CTCAE), version 4.0, at the start of sunitinib therapy; and those with active infections requiring treatment. We will also exclude patients with severe liver injury (ie, alanine aminotransferase ≥5 × upper limit of normal or 2 × individual baseline value); severe kidney injury (ie, serum creatinine level ≥2 × individual baseline value); and other patients who are determined to be inappropriate for study participation by the principal investigator.

### Intervention

The participants will apply three fingertip units of Amitose DGA-containing cream all over both the palmar and plantar surfaces more than three times a day, within two treatment cycles (ie, 6 weeks) of sunitinib. They will also apply heparinoid or urea-containing cream as a standard preventive treatment for HFSR following application of the investigational cream. The participants will be instructed to apply the investigational cream to moist skin, such as when washing their hands or feet, during face washing, or after bathing. The participants will keep a personal diary to record the frequency of application of Amitose DGA-containing cream.

### Study Outcomes

#### Primary Outcomes

The primary outcome of Phase I is development of dermatological abnormalities on the palmar or plantar surfaces, such as bullous dermatitis, dry skin, erythroderma, pruritus, purpura, rash maculopapular, skin hyperpigmentation, and skin hypopigmentation, within two cycles (ie, 6 weeks) of sunitinib therapy. The primary outcome of Phase II is development of grade 1 or higher HFSR within 6 weeks after the initiation of sunitinib therapy. These outcomes will be evaluated by qualified dermatologists.

#### Secondary Outcomes

The secondary outcomes of Phase I are hematological test abnormalities within the observation period. The secondary outcomes of Phase II are development of grade 2 or higher HFSR within 6 weeks of sunitinib therapy, progression-free survival and time-to-treatment failure of sunitinib therapy, dermatological abnormalities of the palmar or plantar surfaces within the observation period, and development of grade 2 or higher HFSR within 3 weeks after completion of the investigational treatment. HFSR and dermatological abnormalities will be evaluated by qualified dermatologists.

### Safety Endpoints

The safety endpoints are dermatological abnormalities in palmar or plantar surfaces, such as bullous dermatitis, dry skin, erythroderma, pruritus, purpura, rash maculopapular, skin hyperpigmentation, and skin hypopigmentation, as well as the frequency and severity of treatment-emergent adverse events. These adverse events have a cause-and-effect relationship with the investigational preparation, so we will also observe them over the whole treatment period.

### Study Procedure

The study schedule is shown in Table 1. Screening tests will be performed within 1 week of obtaining informed consent and researcher confirmation of each patient’s eligibility. Eligible patients will be enrolled via electronic case report forms (eCRFs).

Baseline data will be corrected beginning on the initial day of investigational cream usage. Patient-specific data (ie, sex, height, weight, concomitant diseases, medical history, prior chemotherapy, etc) and hematological and biochemical test data will be acquired from patients’ electronic medical records as per usual care practice information. The baseline dermatological status of each participant will be determined from the screening test.

Patients will receive sunitinib for 2 weeks, followed by a 1-week interruption; therefore, the therapeutic cycle lasts for 3 weeks. The initial dosage of sunitinib will be 37.5 mg/day. The participants will begin treatment with Amitose DGA-containing cream simultaneously with the start of sunitinib therapy. Plasma concentrations of sunitinib will be measured 10-14 days after the start of sunitinib therapy, as per usual care practice. HFSR will be checked regularly by the hospital urologists during the first 2 weeks of sunitinib therapy; during this period, the patients will remain in hospital.

During week 3 and week 6 outpatient clinical visits, participants will receive dermatological examinations by a dermatologist. This examination will focus on dermatological abnormalities of the palmar or plantar surfaces to assess the safety of the Amitose DGA-containing cream in Phase I. The dermatologist will additionally assess the efficacy of the Amitose DGA-containing cream at these visits during Phases I and II.

### Assessments

#### Hand-Foot Skin Reaction

HFSR grading will be done according to that for palmar-plantar erythrodysesthesia syndrome, as described by the National Cancer Institute CTCAE, version 4.0. The dermatologists will then determine the efficacy of the investigational preparation.

**Table 1:** Study schedule of events.

Study events and measurements	Screening phase	Treatment phase^a^						Posttreatment observation phase
	Within 1 week of obtaining informed consent	Week 1	Week 2	Week 3	Week 4	Week 5	Week 6	Week 9
Sunitinib treatment		✓︎	✓︎		✓︎	✓︎		
Application of Amitose DGA cream		✓︎	✓︎	✓︎	✓︎	✓︎	✓︎	
Informed consent	✓︎							
Patients’ backgrounds	✓							
Hematology^b^ and biochemistry^c^	✓			✓			✓	
Sunitinib plasma concentration			✓					
Subjective symptoms	✓	✓		✓			✓	✓
Objective findings	✓	✓		✓			✓	✓
Dermatological examination	✓			✓			✓	
Adherence confirmation		✓	✓	✓			✓	
Observation of adverse events		✓︎	✓︎	✓︎	✓︎	✓︎	✓︎	✓︎

^a^The weeks listed correspond to the weeks after initiation of sunitinib.

^b^Hematology tests include red blood cell counts, hemoglobin, hematocrit, differential leukocyte counts, and platelet counts.

^c^Biochemistry tests include total protein, serum albumin, aspartate aminotransferase, alanine aminotransferase, alkaline phosphatase, gamma-glutamyl transpeptidase, lactase dehydrogenase, total bilirubin, direct bilirubin, creatine kinase, blood urea nitrogen, serum creatinine, uric acid, and serum concentrations of sodium, potassium, chloride, and calcium.

#### Compliance for Use of Investigational Preparation

Participant compliance will be monitored by the medical staff while the participants are inpatients. Once participants are transitioned to outpatient treatment, we will calculate a compliance ratio for the investigational preparation by determining instances of daily use, based on diaries kept by individual participants.

#### Dermatological Abnormalities

Dermatological abnormalities will be defined as grade 1 or higher dermatological events, including bullous dermatitis, dry skin, erythroderma, pruritus, purpura, rash maculopapular, skin hyperpigmentation, and skin hypopigmentation, as specified by the CTCAE, version 4.0. Dose-limiting toxicities will be defined as grade 2 or higher events. If dose-limiting toxicity is more than 40% and is confirmed to be causally connected to the use of the investigational preparation, the study will be terminated and will not advance to Phase II.

#### Plasma Concentration of Sunitinib

Plasma concentrations of sunitinib will be measured 10-14 days after the start of sunitinib therapy, as per usual care practice; the trough level of total concentration of sunitinib and its metabolite N-desethyl-sunitinib will be measured.

#### Progression to Sunitinib Therapy

Before the introduction of sunitinib, all patients will undergo radiological examinations, including computed tomography (CT) imaging of the brain, chest, and abdomen, or radionuclide bone scans, or both. Typically, tumor measurements are performed using CT every 8-12 weeks after initiating sunitinib therapy. All responses will be assessed by a treating physician based on the Response Evaluation Criteria in Solid Tumors, version 1.1.

### Discontinuation of Study Subject Participation

Necessary testing will be carried out to assess the efficacy and safety of the Amitose DGA-containing cream if its use is permanently discontinued due to termination of subject participation in the study. Participants can be withdrawn from the study if their consent is withdrawn; if inadequacies are found after enrollment; if sunitinib therapy is determined to be unnecessary because of RCC resolution; or if the patient is unable to continue sunitinib therapy because of disease progression, complications, or adverse events induced by sunitinib or Amitose DGA-containing cream. Participation can be terminated in cases of pregnancy, noncompliance with the use of the investigational preparation (ie, compliance ratio less than 70%), if the study is discontinued, or if other issues emerge that warrant study discontinuation, according to the physician.

### Statistical Plan

All analyses will be conducted using SPSS Statistics for Windows, version 24.0 (IBM Corp) or later. Interim analyses will be not performed.

#### Sample Size Calculation

Participants were enrolled in the Phase I cohort group until the sample size reached 5 patients, in the event that study discontinuation occurred before the use of the investigational preparation. The sample size for the Phase I study was primarily based on the extent of necessity and concernment. To limit the potency of intolerable adverse events, the investigational preparation is an ascorbic acid derivative, which is generally equivalent to cosmetic preparations; however, there are no practical safety data pertaining to the administration of Amitose DGA-containing cream to patients with RCC receiving sunitinib therapy. In this study, the safety of Amitose DGA has been confirmed by a cohort of 5 patients.

The sample size for the Phase II study will be 30 participants, in combination with the Phase I study sample (ie, 5 Phase I participants and 25 additional participants). In previous reports from our institution, HFSR of any grade (ie, grades 1-3) was 33.3%; HFSR of grade 3 was 2.2% among patients with metastatic RCC who were treated with a 2-week-on and 1-week-off sunitinib schedule [18]. Given that the investigational preparation can prevent up to 75% of grade 1-2 HFSR, we estimate that the frequency of HFSR of any grade (ie, grades 1-3) among patients using the investigational preparation will be 10%. In the case of this study, 25 participants will be needed to guarantee an alpha of .05 and 80% statistical power, with no continuity correction. Therefore, we aim to recruit 30 participants to mitigate potential exclusions from the analysis set.

#### Primary Analysis

In the Phase I study, the safety of the investigational preparation will be evaluated at the end of two cycles of sunitinib therapy. If 2 or more participants out of 5 (≥40%) show dose-limiting toxicities during the above safety evaluation period or if the fifth participant is confirmed to have started the investigational preparation normally, ongoing Phase I enrollment will stop. At this time, the study secretary and principal investigator will discuss and determine the safety of the investigational preparation, based on the eCRF that records safety evaluation data.

The null hypothesis of the Phase II study has been defined as the frequency of development of HFSR of any grade (ie, grades 1-3) is 33.3% [18], and the frequency of development of HFSR of any grade (ie, grades 1-3) in one sample is analyzed with a significance level of 5%.

#### Secondary Analysis

Secondary analysis on the efficacy of the investigational preparation throughout the whole observation period will be performed for additional discussion on the primary analysis, but multiplicity will not be adjusted in this analysis. Hypothesis testing will be performed with a two-sided 5% significance level and a two-sided 95% CI.

Logistic regression analysis, setting the development of HFSR as the dependent variable, will be performed by examining independent variables, including patient characteristics, sunitinib dose, and plasma concentrations of sunitinib, with a two-sided significance level of 5% and a two-sided 95% CI. Moreover, progression-free survival and time-to-treatment failure will be determined using the Kaplan-Meier estimate; medians and 95% CIs will be calculated.

We will carry out imputation for missing data by multiple imputation, as appropriate. Primary analyses will be performed by intention-to-treat analysis. Secondary analyses will consider per-protocol set, including participants who completed the treatment according to the scheduled protocol.

### Pharmacovigilance and Data Monitoring

We will pay for the management of all serious adverse events suffered by subjects in the clinical trial and will compensate them or their families for injuries or deaths related to the study, using the clinical trial insurance coverage. All serious adverse events will be reported to the principal investigator within 24 hours of the trial investigator becoming aware of each event. Subsequently, the principal investigator will report each event to the ethics committee within 48 hours. All relevant information about any suspected or unexpected serious adverse reactions that occur during the study, particularly those that are fatal or life-threatening, will be reported as soon as possible, and no later than 7 days, to the appropriate authorities.

The principal investigator will designate a monitor to review individual subject safety data in an ongoing fashion and will monitor the data collected throughout the study, thus providing quality control for the study. Because of the small size of this study, an audit is not planned.

### Privacy and Confidentiality

Both the eCRFs and the personal computer storing the eCRFs will be password protected. The computer will be stored in a secure location under the care of the study secretary, and the eCRFs will be destroyed after study completion. Privacy and confidentiality will be further secured by assuring that only deidentified data will be used in place of personal identifiers within all eCRFs.

## Results

This study is ongoing. Recruitment to the study began in August 2017 and is ongoing in Japan. To date, 21 subjects have been recruited. Study completion is expected in 2021. The results of the study will be disseminated through one or more scientific papers and may also be presented at medical conferences. The datasets generated and the data analyzed during this study will be available from the corresponding author upon appropriate request after publication.

## Discussion

HFSR is not a life-threatening side effect of multiple-TKIs but can drastically decrease quality of life and adherence to chemotherapy. This is the first clinical study of an Amitose DGA-containing cream in patients with RCC receiving sunitinib therapy. Moreover, this may be the first clinical study to use cosmetic preparations to determine dermatological adverse events induced by anticancer agents. This study will inform future, novel, prevention methods for HFSR.

We have reported the effects of ascorbic acid derivatives on keratinocyte toxicity induced by multiple-TKIs in vitro [13]. We verified these effects using an animal-testing alternative, consisting of a reconstructed human epidermal model in vitro. This methodological approach has also been used to evaluate dermatological adverse events [19-21]. Our experiment was conducted using similar methods; however, our results are preliminary and require further validation. Considering the evidence from our previous study, we aimed to evaluate both the effects of ascorbic acid derivatives alone on HFSR and the effects of combination therapy involving ascorbic acid derivatives and an existing standard-of-care prophylaxis.

The subjects of this study are patients receiving sunitinib therapy, not including other multiple-TKIs. We selected a single-arm, open-label design to maximize subject exposure and increase the likelihood of achieving study endpoints. Given our limited study population, the results of this study will be clear but the insights gleaned will be limited in their potential application.

Results from our previous study indicated that sunitinib-induced HFSR is likely to develop within 6 weeks of sunitinib initiation [22]. In this study, HFSR evaluated at weeks 3 and 6 was considered induced by sunitinib. Because sunitinib-induced HFSR also develops in latter phases, longer observation periods should be considered. Because adverse events related to sunitinib can cause dose reduction and interruption [23,24], the primary endpoint was set within two therapeutic cycles, considering study feasibility and clarity of results.

The exclusion criteria were established to assist with the safe and ethical performance of this study. These criteria differed from the exclusion criteria of a previous study, which reported on the frequency of HFSR with a 2-week-on and 1-week-off schedule of sunitinib conducted at our hospital [18]. The criteria for this study were set in order to compare the development of HFSR; we do not believe this will adversely affect subject recruitment.

Development of HFSR is associated with higher plasma concentrations of sunitinib [25-27]. Therefore, our secondary endpoints include the evaluation of plasma concentrations of sunitinib around day 10, as this time point is believed to represent steady-state concentrations [28].

This study will provide preliminary and valuable evidence to support the use of Amitose DGA in the prevention of HFSR. Amitose DGA may improve the pain and suffering caused by therapy with multiple-TKIs, while enhancing therapeutic outcomes.

## Abbreviations

Amitose DGA: bis-glyceryl ascorbate
CT: computed tomography
CTCAE: Common Terminology Criteria for Adverse Events
eCRF: electronic case report form
HFSR: hand-foot skin reaction
P-VC-Mg: ascorbyl-2-phosphate magnesium
RCC: renal cell carcinoma
TKI: tyrosine kinase inhibitor
